# Accuracy and distribution of baseline categorical variables and *p*-values in spine randomized controlled trials

**DOI:** 10.1098/rsos.240170

**Published:** 2025-01-15

**Authors:** Mark J. Bolland, Alison Avenell, Andrew Grey

**Affiliations:** ^1^ Department of Medicine, University of Auckland, Private Bag 92 019, Auckland 1142, New Zealand; ^2^ Department of Endocrinology, ADHB, Private Bag 92 024, Auckland 1142, New Zealand; ^3^ Health Services Research Unit, University of Aberdeen, Foresterhill, Aberdeen AB25 2ZD, UK

**Keywords:** spine, randomized controlled trials, *p*-values, randomization, statistical tests

## Abstract

Levayer and colleagues assessed integrity issues in randomized controlled trials (RCTs) in four spine journals using baseline *p*-values from categorical variables, concluding that there was no evidence of ‘systemic fraudulent behaviour’. We used their published dataset to assess the accuracy of reported *p*-values and whether observed and expected distributions of frequency counts and *p*-values were consistent. In 51 out of 929 (5.5%) baseline variables, the sum of frequencies did not agree with the reported number of participants. For one-third of reported *p*-values (172 out of 522), we could not calculate a matching *p*-value using a range of statistical tests. Sparse data were common: for 22% (74 out of 332) of variables in which the reported *p*-value matched the *p*-value calculated from a chi-square test, the expected cells were smaller than recommended for the use of chi-square tests. There were 20–25% more two-arm trials with differences in frequency counts of 1 or 2 between-groups than expected. There were small differences between observed and expected distributions of baseline *p*-values, but these depended on analysis methods. In summary, incorrectly reported *p*-values and incorrect statistical test usage were common, and there were differences between observed and expected distributions of baseline *p*-values and frequency counts, raising questions about the integrity of some RCTs in these journals.

## 1. Introduction

Levayer and colleagues surveyed 167 randomized controlled trials (RCTs) in four selected ‘spine’ journals for evidence of non-random baseline frequency data, concluding that there was no widespread evidence of data fabrication or non-random allocation [1]. The authors should be congratulated for such a major undertaking and for making data available with their publication. To our knowledge, this is the only published dataset of baseline categorical variables from a large group of RCTs.

We have previously reported that baseline categorical data can be included in the assessment of publication integrity of groups of RCTs [2–4], and have published a freely available package ‘reappraised’ for the R statistical software program (https://CRAN.R-project.org/package=reappraised). We took the opportunity to assess the dataset created by Levayer *et al*. [1] using the tools in the reappraised package.

The purpose of the study was first to replicate the work of Levayer and colleagues. Second, we wished to extend their approach and see whether their conclusions held if more assessments were undertaken. Levayer and colleagues assessed a single aspect of the RCTs: whether the distribution of *p*-values from the comparison of baseline categorical variables using a *χ* ^2^-test was consistent with valid randomization using the Stouffer method. This approach produces a single *p*-value for each RCT, and *p*-values <0.05 or >0.95 were considered potentially inconsistent with randomization because the baseline variables were too similar or dissimilar. We added a number of other assessments: whether the reported number of participants by group for each baseline variable matched the number of randomized participants; whether the reported baseline *p*-values matched those we calculated independently; and third, whether the distribution of baseline variables and *p*-values was consistent with expected distributions. Finally, we considered whether the conclusions of Levayer and colleagues remained valid with these additional assessments.

## 2. Methods

We imported the published Excel spreadsheets [1] into R and assembled them into a single data frame. This generated a dataset with 167 studies and 940 variables, of which 11 variables were ignored or excluded by Levayer and colleagues, leaving 929 variables for analysis—the ‘spine dataset’. In the originally published dataset, there are 924 variables in the summary table (column ‘nb of bin variables’), but Levayer and colleagues state they used 921 in their analysis [1]. The difference of five variables between the spine dataset we created and the summary table was one variable with a reported and calculated *p*-value was not analysed; one variable with a reported *p*-value but no calculated *p*‐value (and a large difference between the sum of frequencies and the number of participants) was not analysed; and three variables with only one level and with no reported or calculated *p*-values and either 0 or 100% frequency for the solitary level were not analysed. We included all five variables in the spine dataset.

Chi-square tests are not appropriate in the presence of sparse data. We used the commonly applied rule of at least one cell with an expected value <5 for 2 by 2 tables and at least one cell with an expected value of <1 or >20% of cells with an expected value of <5 for tables larger than 2 by 2 as the thresholds for indicating sparse data such that the expected cells are too small for the *χ* ^2^ test and that an exact test should be used. When Fisher’s exact test is performed on tables larger than 2 by 2, the computational memory required can become very large with long running times. We used 10 000 Monte-Carlo simulations in the R fisher.test function to avoid this issue.

We checked whether the number of participants for each variable by group matched the reported number of participants in the entire RCT. Next, we used the cat_all_fn (reappraised package, R) to independently calculate the *p*-value for the comparison of baseline categorical variables using a range of statistical tests and check whether any of the calculated *p*-values matched the reported value. Next, we used the cat_all_fn (reappraised) to check whether the observed distributions of categorical variables were consistent with the expected distributions. Finally, we used the pval_cat_fn (reappraised) to assess the distribution of calculated baseline *p*-values compared to the empirically calculated distribution (i.e. the distribution of *p*-values in a large number of simulated datasets based on the reported summary statistics for each categorical variable and assuming the variables were binomially distributed). All analyses were conducted using R software packages (R 4.2.0, 2022, R Foundation for Statistical Computing, Vienna, Austria).

## 3. Results

### 3.1. Baseline variables

For 51 out of 929 (5.5%) variables, the sum of frequencies by level for each variable did not agree with the reported total number of participants. For 11 (1.2%) variables, the sum of frequencies exceeded the total number of participants, implying data extraction errors by Levayer and colleagues, typographical errors by either Levayer and colleagues or the trial authors or impossible data. For example, one study had nine smokers and 38 non-smokers (47 total), but only 43 participants. Five of the 11 differences were 1, another three were <10 and three were >10 (per cent difference range 0.4–32%). For a further 40 (4.3%) variables, the sum of frequencies for each variable was smaller than the total number of participants, again implying missing data or errors such as data extraction, typographical or other errors. Thirty-one out of 167 (19%) of studies had variables with either of these issues and 12 (7%) studies had both.

### 3.2. Comparing reported and calculated baseline *p*-values

Levayer and colleagues extracted *p*-values from the original publications for the comparison between groups (baseline *p*-values). Out of 871 baseline variables, 166 studies reported 495 *p*-values (the reported *p*-values), with the sum of the frequencies equalling the number of participants and at least one frequency count >0. The statistical test used to generate the *p*-values in the original publications was not described in the spine dataset. For these 495 variables, 481 had a numeric *p*‐value. We independently calculated the *p*-value for each variable from the summary statistics in the spine dataset and rounded it to the same number of decimal places as the reported *p*‐value. Table 1 shows that 328 (68%) reported *p*-values matched with a calculated *p*‐value (217 *χ* ^2^ test, three *χ* ^2^ test with continuity correction, 100 Fisher’s exact test, four mid-*p* test and four other tests). Of the 153 variables (32%) with mismatched reported and calculated *p*-values, the difference between the reported *p*-value and the closest calculated *p*-value ranged from 0.0001 to 1 (median difference 0.05, interquartile range 0.01–0.16). Of the 14 variables with a reported threshold *p*‐value (*p* > 0.05, >0.99, >0.999 or >0.9999), 11 out of 14 (79%) had a matching calculated *p*‐value. The three non-matching values were reported *p*‐value >0.9999 and closest calculated *p*-values were 0.69, 0.86 and 0.86, respectively. Overall, mismatched reported and calculated *p*-values were present in 62 out of 166 (37%) studies.

**Table 1.** Difference between reported and calculated baseline *p*-values.

		baseline *p*‐value									
difference between reported and calculated *p*-values	*n* (%)	0– 0.1	0.1–0.2	0.2–0.3	0.3–0.4	0.4–0.5	0.5–0.6	0.6–0.7	0.7–0.8	0.8–0.9	0.9–1
0	328 (68)	31	22	31	26	43	31	37	28	34	45
0–0.1	96 (20)	9	5	11	5	14	11	9	10	8	14
0.1–0.2	29 (6)		1	2	4	4	9	1	4	1	3
0.2–0.3	8 (2)		1			2	3		2		
0.3–0.4	8 (2)		3			1	1	3			
0.4–0.5	5 (1)			1	2	1				1	
0.5–0.6	2 (0)			1			1			
0.6–0.7	4 (1)	1	1					1	1		
0.9–1	1 (0)										1

There were 220 variables that had a matching calculated *p*-value from a *χ* ^2^ test. Of these, 44 (20%, 20 from 2 by 2 tables, 24 from tables larger than 2 by 2) had expected cells that were too small for the *χ* ^2^ test to be an appropriate choice (i.e. data were sparse). Overall, 74 out of 332 (22%) *χ* ^2^ tests in 51 out of 167 (30%) studies were done even though the data were sparse.

We repeated the analyses for the 51 variables in which the frequency totals did not agree with the total participants, using the reported frequency counts. Twenty-five (45%) had a reported *p*-value and all were numeric. Ten (40%) of the reported *p*-values had a matching calculated *p*‐value (eight *χ* ^2^ test, two Fisher’s exact). The differences for non-matching *p*-values ranged from 0.002 to 1 (median difference 0.03, interquartile range 0.006–0.12). Eight out of 17 (47%) *χ* ^2^ tests had expected cells that were too small (six tables larger than 2 by 2 and two 2 by 2 tables), in seven different studies.

Finally, there were seven variables in which all the frequency counts were either 0 or the total number of participants in each group. For these variables, a test on a 2 by 2 table cannot be performed. However, a one-way *χ* ^2^ test can be done. Two variables had a reported *p*‐value (both 1). Only one of the reported *p*-values matched that obtained from a one-way *χ* ^2^ test (1 and 0.06, respectively).

In the entire spine dataset, there were 522 variables with reported *p*-values. For 172 (33%) of these reported *p*-values, we could not calculate a matching *p*-value using a range of standard statistical tests. Eighty-two out of 522 variables (16%) had expected cells that were too small for the *χ* ^2^ test to be an appropriate choice and of these, 47 had a reported *p*-value that matched with a *p*-value calculated with a *χ* ^2^ test.

### 3.3. Distribution of frequency counts

**Figure 1.**
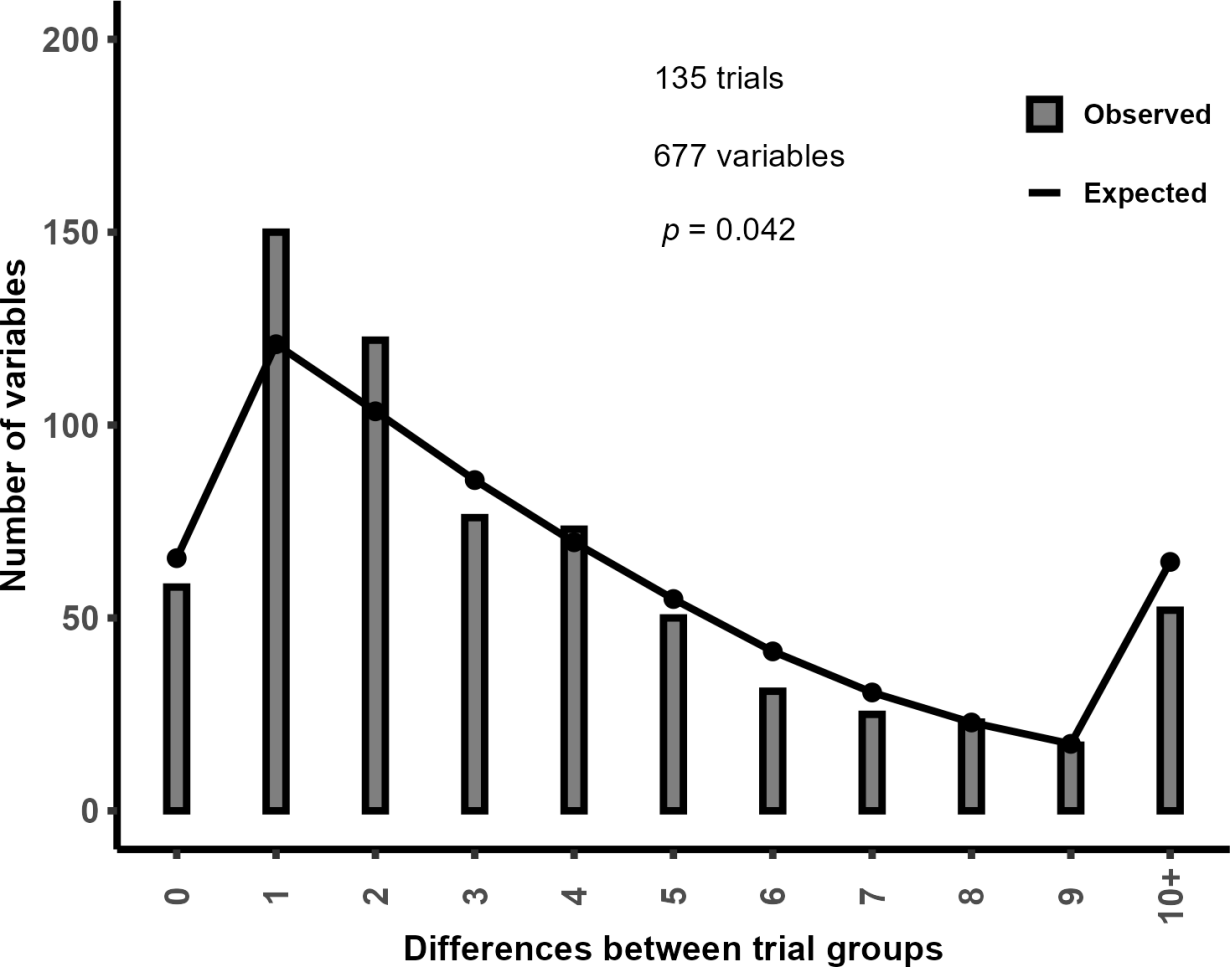
Distribution of observed and expected differences in frequency counts between groups in two-arm trials.

There were 135 two-arm trials with 677 variables, for which the sum of the frequency counts matched the total participants. We assessed the differences between groups for frequency counts in these trials. We randomly excluded a single level to avoid double counting (for example, if gender was reported as male or female, we randomly selected one level because the other level is redundant and can be calculated from the total number of participants and the selected level). Figure 1 shows that there was an excess of variables with a difference of 1 or 2 compared with the expected distribution. The differences between the observed and expected differences of 1 and 2 were 29 and 18, respectively. When we included variables in which the total frequency counts differed from the total participants, there were 138 trials and 713 variables and the results were similar.

### 3.4. Distribution of baseline *p*-values

Finally, we assessed the distribution of calculated baseline *p*-values compared with the expected distribution. This was calculated empirically from 100 simulated datasets in which each variable for each individual was randomized to a treatment group, creating 100 simulated datasets in which each variable and simulation had been independently randomized. We performed analyses separately on variables where the sum of the frequency counts agreed with the total number of participants and variables where they did not agree. We present results for variables where they agreed, although the results were similar for the analyses of all variables. The results were heavily influenced by the choice of statistical test because many of the variables contained sparse data where the expected cells were too small for the *χ* ^2^ test (294 out of 922 (32%) variables in spine dataset without frequency count of 0 or 100%, 134 out of 642 (21%) 2 by 2 tables; 160 out of 280 (57%) larger than 2 by 2 tables). Levayer and colleagues used *χ* ^2^ tests for all variables in their primary analyses [1]. However, they noted the issues with sparse data and used mid-*p* tests to analyse one small study in a secondary analysis [1]. For tables with more than two rows or columns, Fisher’s exact test is the only easily accessible option for sparse data.

**Figure 2.**
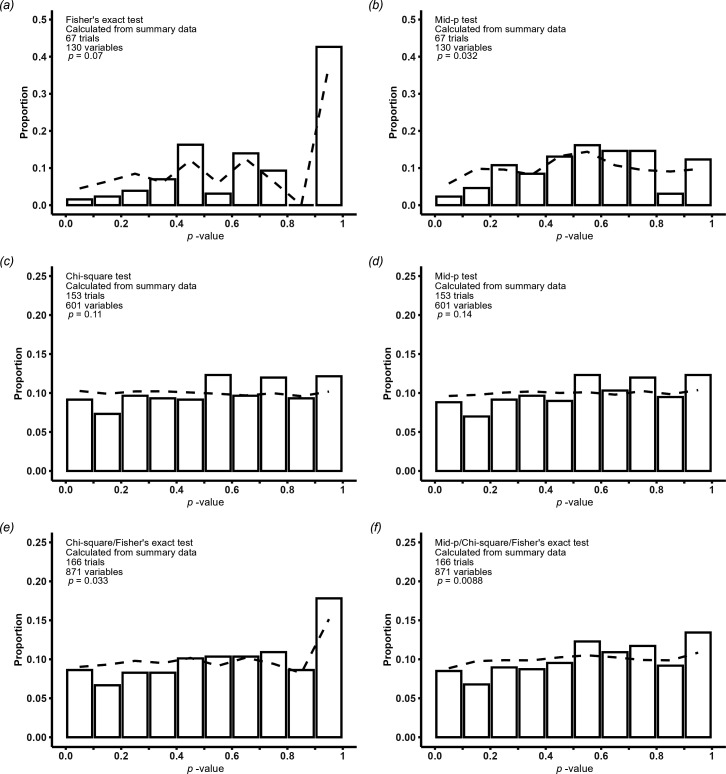
Observed (bars) and empirically calculated expected (dotted line) distribution of baseline *p*-values. (*a*) calculated using Fisher’s exact test and (*b*) using a mid-*p* test in variables with only two levels and two treatment groups (2 by 2 table) with sparse data. (*c*) calculated using a *χ* ^2^ test and (*d*) using a mid-*p* test for 2 by 2 tables and a *χ* ^2^ test for larger tables in variables without sparse data. (*e*) calculated using *χ* ^2^ tests for variables without and Fisher’s exact test for variables with sparse data and (*f*) using mid-*p* tests for 2 by 2 tables and *χ* ^2^ (without sparse data) or Fisher’s exact test (with sparse data) for larger tables. All figures are for variables where the sum of frequency counts agreed with the participant numbers.

Figure 2*a*,*b* shows the distribution of *p*-values in variables which had two treatment groups and two levels (i.e. 2 by 2 table) with sparse data. They show that the distribution of *p*-values, both observed and expected, differs markedly depending on whether mid-*p* or Fisher’s exact test are used. The observed distribution differed from the expected distribution for the mid-*p* test but was similar for the Fisher’s exact test. Figure 2*c*,*d* shows that the observed and expected distributions were similar for *p*-values from variables without sparse data when calculated with the *χ* ^2^ test or the combination of mid-*p* test for 2 by 2 tables and *χ* ^2^ test for larger tables. Figure 2*e*,*f* shows the distribution of *p*-values in all variables using either *χ* ^2^ tests for variables without sparse data and Fisher’s exact test for variables with sparse data or mid-*p* tests for 2 by 2 tables and *χ* ^2^ (without sparse data) or Fisher’s exact test (with sparse data) for larger tables. For both analyses, the observed and expected distributions were visually fairly similar but there were statistically significant differences between them.

**Figure 3.**
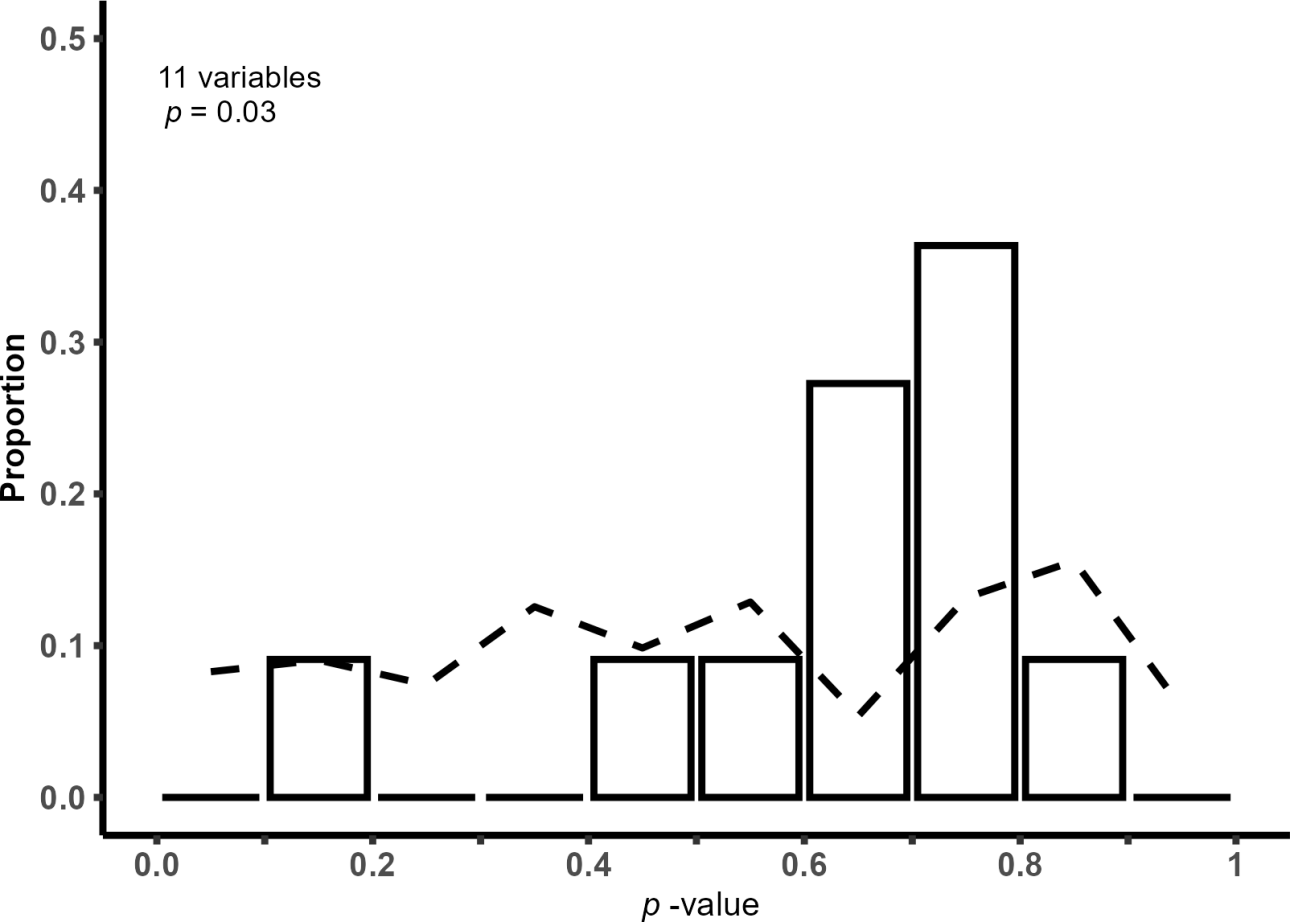
The observed (bars) and expected (dotted line) in a single small study for which mid-*p* tests were used to calculate baseline *p*-values.

The issue regarding statistical tests is illustrated well by an example from the paper by Levayer *et al*. [1]. A trial with 30 participants [5] reported 11 variables and baseline *p*-values were calculated using the *χ* ^2^ test to give a studywise *p*-value of 0.996. Seven out of 11 variables had expected cells <5. The baseline *p*-values were recalculated using a mid-*p* test, giving a studywise *p*-value of 0.92, which the authors said was reassuring. However, the actual distribution of mid-*p*-values was unusual—7 out of 11 mid-*p*-values were between 0.6 and 0.8 and 10 out of 11 between 0.49 and 0.85. The expected distribution calculated empirically was a ‘noisy approximately uniform’ distribution, as shown in figure 3 and the observed distribution differed markedly from this.

## 4. Discussion

### 4.1. Implications for the reliability of trials in spine journals

In these analyses, we were unable to independently replicate the *p*-value calculation for about one-third of variables with a reported baseline *p*-value that had been extracted by Levayer and colleagues. Of those we could replicate, about 1 in 5 used a *χ* ^2^ test even though there were sparse data with expected cells too small for the *χ* ^2^ test to be an appropriate choice, indicating that an exact test (e.g. mid-*p* or Fisher’s exact test) should be considered. In two-arm trials, there was a 20–25% excess of variables in which the between-groups difference in frequency counts was 1 or 2 compared with the expected amount. Similar findings have been reported in groups of trials with integrity concerns [4]. There were small differences between the observed and empirically calculated distributions of baseline *p*-values, particularly for those with sparse data. However, the importance of these findings remains uncertain. Collectively, these issues raise concerns about the reliability of some of the trials in this dataset.

### 4.2. Comparison to the previous results

Levayer and colleagues concluded that there was no evidence of systemic fraudulent behaviour or non-random allocation in these trials [1]. They commented that their approach ‘will detect fraud, provided it presents as non-random allocation.’ They therefore suggested that readers can ‘generally assume that RCTs published in these spine journals are genuine’ [1].

We think that any suggestions of fraud based simply on the assessment of data in publications without an investigation should be avoided because such assessments can only determine the existence of issues such as errors, impossible or improbable data, plagiarism, image manipulation and statistical patterns inconsistent with randomization [6]. These issues can be grouped under the term publication integrity, which can be readily assessed. However, if compromised integrity is identified, the reasons for the problems (such as honest error or questionable research practices, including fraud or fabrication) can only be determined from an investigation [6]. The reason for the compromised integrity matters little to the readers of the publications, whose main concern is that the publication is reliable. For these reasons, we think it is unwise to make any inferences about the presence or absence of fraud in these trials.

Putting that important issue aside, there are a number of other reasons to be cautious. First, 167 trials were assessed, but 11 had only a single baseline *p*‐value and 98 (59%) had five or fewer *p*-values. The number of *p*-values in a study needed to draw a reliable conclusion from analyses of baseline *p*-values is not known, but it is likely to be considerably higher than five unless the *p*-values are extremely skewed. Three out of 69 (4%) and 2 out of 10 (20%) trials with at least 5 or 10 baseline *p*-values, respectively, were identified using the threshold of a studywise *p*-value of >0.99 and a further five trials with >5 baseline *p*-values using the thresholds of <0.05 or >0.95. One of the three trials exceeding the 0.99 threshold has been retracted. This seems less reassuring than Levayer and colleagues’ conclusion.

Furthermore, the test the authors used, the Carlisle–Fisher–Stouffer method, only detects situations where there is an excess of baseline *p*-values close to 0 or close to 1. Carlisle made it clear that he was looking for outliers: trials that did not conform to this test of baseline data [7]. Carlisle was not seeking to detect every case of compromised data [7]. Outlying results might have many explanations, one of which and perhaps the least likely, is fabricated data. An example where this technique does not work well is that a study could have 10 baseline *p*-values between 0.45 and 0.55, a very unlikely distribution, but the studywise *p*-value would be close to 0.5. In addition, Carlisle used the technique for continuous data and to our knowledge, it has not been applied to categorical data previously. Categorical data differ from continuous data in that the baseline *p*-values are not uniform and, particularly when the sample size is small, there are only a small number of discrete *p*-values [2]. The combination of small sample sizes and sparse data means a high proportion of *p*-values calculated using Fisher’s exact test will be 1 (figure 2*a*). However, Levayer and colleagues chose to use the *χ* ^2^ rather than exact tests, which may have impacted the studywise *p*-values calculated. If there is a baseline *p*-value of 1, the *z*-score used in the Carlisle–Fisher–Stouffer method cannot be derived. Levayer and colleagues chose to substitute a value of 3, comparable to a *p*-value of 0.998, including for the situation where all the frequency counts were 0 (e.g. all or none of the participants in the trial had the characteristic). It is not certain what the best approach should be for these situations; possibilities could include excluding sparse data, calculating one-way *p*-values for variables with frequency counts of 0 or using different statistical tests. However, if different statistical tests are used, the baseline *p*-value distribution may change substantially but remain different from the expected distribution (figures 2 and 3). A recent preprint has proposed a solution that may address some of these issues [8].

### 4.3. Broader implications

The analysis by Levayer and colleagues and our replication assessed only a very small spectrum of the scientific literature: RCTs in four journals in the narrow field of spine research. It is not known how generalizable these findings might be, both to RCTs in other fields or to other study designs. Studies like that of Levayer and colleagues, in which large amounts of baseline data are extracted from large numbers of publications, are extremely time-consuming, laborious and infrequent. Surveys of publications from individual research groups have been carried out as part of the assessment of publication integrity and reported that the groups of publications had patterns of results in the baseline variables that differ from the expected patterns [9–12]. Others have reported that errors are commonly found by experienced investigators with relevant expertise simply by reading papers while keeping abreast of articles in their field [13]. In a monumental piece of research, Carlisle extracted data on 29 789 continuous baseline variables in 5087 RCTs published in eight journals over 15 years, reporting that about 5% more RCTs than expected had baseline *p*-values that were highly similar or dissimilar [7], often owing to reporting errors. Of 100 clinical trials that were closely examined, 42 out of 50 (84%) retracted trials had mathematical or factual discrepancies, but so did 24 out of 50 (48%) unretracted trials [14]. Taken together, it seems likely that a substantial proportion of publications on RCTs have errors or other problems. The consequences of those errors might be trivial, for example, a typographical error in a table or they may completely alter the conclusions [15]. Whether similar findings would occur in other research designs or disciplines is not known, but such transdisciplinary research would be valuable.

Retractions of publications with compromised publication integrity are increasing in frequency and in 2022 accounted for more than 2 out of 1000 (0.2%) articles published [16]. The implications of our analysis and others reporting frequent errors in publications, along with the increasing retraction rates, suggest that journals and publishers should be implementing strategies to detect and prevent errors and compromised publication integrity before any article is published. Independent statistical and methodological expertise is likely to be valuable in the assessment of publication integrity.

### 4.4. Limitations

We relied on the publicly available dataset and did not compare these data with the original publications, so some of the issues identified might be owing to data extraction or typographical errors. The dataset did not contain the statistical test used to generate or report the *p*-values in the original trials. However, the reappraised package function calculates *p*-values using seven commonly used statistical tests, allowing comparisons to results from all the major statistical approaches. While we have identified some issues regarding the publication integrity of this broad group of trials, we cannot make comments about individual RCTs since we have not assessed any individual trial in detail and because some of the assessments (*p*-value distribution and frequency distribution) were based on the whole group of trials, preventing inferences about individual RCTs. Finally, we only assessed categorical data in the spine dataset. Continuous variables provide a number of options for analysis, which may be preferable to analysing categorical variables [2].

### 4.5. Summary

The dataset published by Levayer and colleagues has allowed a more detailed analysis of baseline categorical data in 167 RCTs in spine journals. This showed that incorrectly reported *p*-values and incorrect usage of statistical tests are common, and that there are differences between the observed and expected distributions of both frequency counts of variables and baseline *p*-values. Collectively, these findings raise questions about the reliability of some spine RCT publications.

A simple potential fix that might prevent some of these issues would be for journals to require authors to provide a table at submission that contains baseline summary continuous and categorical data and *p*-values for the trial in a standardized format that would permit automated extraction and examination of data. Tables 2 and 3 give a worked-illustrative example, and box 1 contains the very simple R code that automatically produces the output. The code could easily be adapted and improved to perform further checks, handle different data or produce different output. If problems are identified, explanations could be sought during the review process for incorrect baseline *p*-values or unusual distributions or matching of baseline data. Publishing this table as electronic supplementary material might be useful because, for example, reporting baseline *p*-values is considered illogical and unnecessary by many experts [21]. Journals should also ensure that the statistical tests applied to categorical variables conform to recommended practice. Independent analysis and publication of anonymized individual patient data is likely to be much more informative for the assessment of publication integrity [22].

**Table 2.** An illustrative example of a baseline table of categorical and continuous variables with errors assembled from different sources. (Abbreviations: *N*, number of participants; *M*, mean; *S*, s.d.; test, statistical test (chisq; chi-square test, *t*-test; Student’s *t*-test); total, total cohort; cat, categorical variable; cont, continuous variable; NS, not stated. Blank squares indicate data not reported.)

			total cohort			group 1			group 2				
variable [reference]	level	type	*N*	*M*	*S*	*N*	*M*	*S*	*N*	*M*	*S*	*p*	test
**categorical variables**													
radiculopathy [5]		total	30			15			15				
radiculopathy	yes	cat	15			6			7				
radiculopathy	no	cat	15			7			8				
level of single-level fusion [17]		total	69			33			36				
level of single-level fusion	L2−3	cat				3			1			0.694	NS
level of single-level fusion	L3−4	cat				8			8			0.694	NS
level of single-level fusion	L4−5	cat				17			37			0.694	NS
level of single-level fusion	L5–S1	cat				5			12			0.694	NS
fracture classification [18]		total	24			12			12				
fracture classification	A3.1	cat				8			7			0.203	chisq
fracture classification	A3.2	cat				4			5			0.203	chisq
**continuous variables**													
ionized calcium [19]		total	62			20			42				
ionized calcium		cont	62	1.23	0.04	20	1.32	0.05	42	1.21	0.05		
left step time [20]		total	20			10			10				
left step time		cont				10	0.65	0.03	10	0.66	0.02	0.624	*t*‐test

**Table 3.** Output from simple checking code and interpretation. (Abbreviations: diff, difference; calc, calculated; NS, not stated; warning, the expected number of observations in each cell is less than the recommendation for a chi-square test; chisq, chi-square test.)

check group numbers					­	­	­	­	­	­	­	­	­	­	­	­	­	­	­
variable	flag	*N*	*n*1	*n*2															
radiculopathy		30	15	15															
level of single-level fusion		69	33	36															
fracture classification		24	12	12															
ionized calcium		62	20	42															
left step time		20	10	10															

check continuous data																			
variable	flag	*M*	*S*	*m*1	*s*1	*m*2	*s*2	*p*	*p* calc	diff *p*-values	mean calculated from subgroups	s.d. calculated from subgroups	diff mean	diff s.d.					
ionized calcium	yes	1.23	0.04	1.32	0.05	1.21	0.05		<0.001		1.25	0.05	yes	yes					
left step time	yes			0.65	0.03	0.66	0.02	0.624	0.392	yes									

check categorical data																			
variable	level	flag	*N*	*n*1	*n*2	*p*	test	*N* total	*N*1	*N*2	*p* calc	warning	diff *p*-values	calc *N*	diff *N*	calc *n*1	diff *n*1	calc *n*2	diff *n*2
radiculopathy	yes	yes	15	6	7			30	15	15	0.978			28	yes	13	yes	15	
radiculopathy	no	yes	15	7	8			30	15	15	0.978			28	yes	13	yes	15	
level of single-level fusion	L2−3	yes		3	1	0.694	NS	69	33	36	0.188	warning	yes	91	yes	33		58	yes
level of single-level fusion	L3−4	yes		8	8	0.694	NS	69	33	36	0.188	warning	yes	91	yes	33		58	yes
level of single-level fusion	L4−5	yes		17	37	0.694	NS	69	33	36	0.188	warning	yes	91	yes	33		58	yes
level of single-level fusion	L5–S1	yes		5	12	0.694	NS	69	33	36	0.188	warning	yes	91	yes	33		58	yes
fracture classification	A3.1	yes		8	7	0.203	chisq	24	12	12	0.673	warning	yes	24		12		12	
fracture classification	A3.2	yes		4	5	0.203	chisq	24	12	12	0.673	warning	yes	24		12		12	

interpretation																			
group numbers	there are no flags for errors in group numbers																		
continuous data	ionized calcium has impossible data—the mean calculated from the subgroup means does not match the reported mean for the cohort																		
	*p*-value calculated from reported data for left-step time differs markedly from reported *p*-value and difference is unlikely to be owing to use of rounded summary data																		
categorical data	group numbers for radiculopathy sum to less than the reported total number of participants																		
	for level of fusion, the sum of group numbers exceeds the reported total number of participants, it is likely a chi-square test was used but there are sparse data and the reported *p*-value does not match the calculated *p*‐value																		
	for fracture classification, the calculated *p*-value does not match the reported *p*-value and sparse data are present

Box 1.
**Simple R code to check standardized baseline table.**

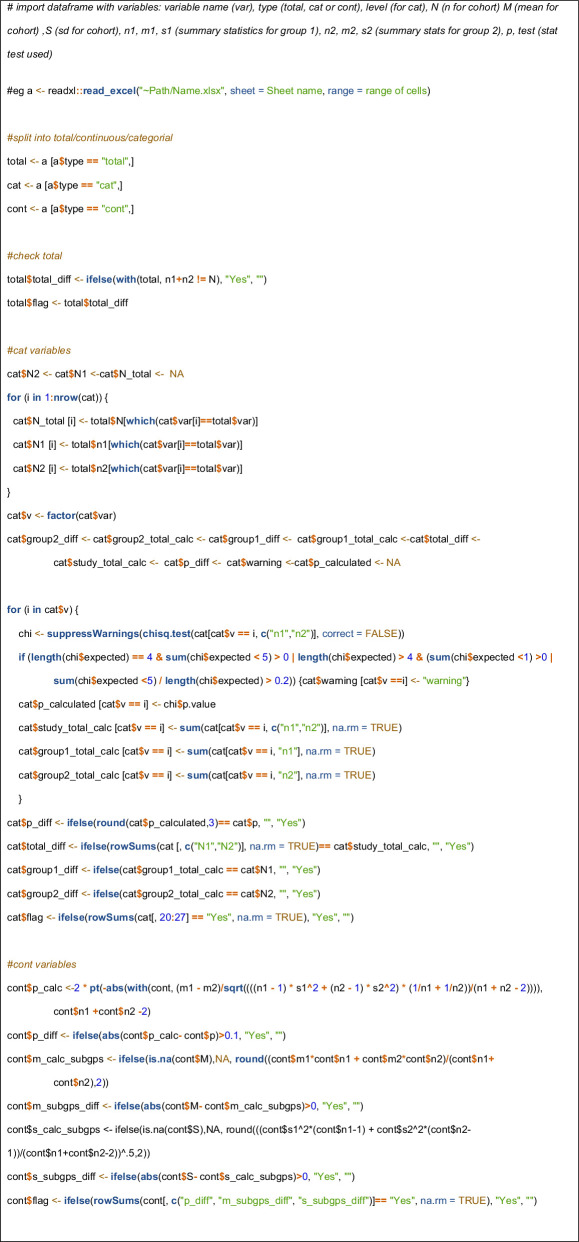

## Data Availability

All data are available as supplementary data to Reference [1]; https://static-content.springer.com/esm/art%3A10.1007%2Fs00586-023-07813-2/MediaObjects/586_2023_7813_MOESM2_ESM.xlsx).
